# 高效液相色谱-串联质谱法同时检测血液中3种季铵盐类肌松剂

**DOI:** 10.3724/SP.J.1123.2020.09020

**Published:** 2021-07-08

**Authors:** Yongpeng HUANG, Hui TANG, Yunyang SONG, Bo CHEN, Hui ZHONG

**Affiliations:** 国民核生化灾害防护国家重点实验室, 北京 102205; State Key Laboratory of NBC Protection for Civilian, Beijing 102205, China; 国民核生化灾害防护国家重点实验室, 北京 102205; State Key Laboratory of NBC Protection for Civilian, Beijing 102205, China; 国民核生化灾害防护国家重点实验室, 北京 102205; State Key Laboratory of NBC Protection for Civilian, Beijing 102205, China; 国民核生化灾害防护国家重点实验室, 北京 102205; State Key Laboratory of NBC Protection for Civilian, Beijing 102205, China; 国民核生化灾害防护国家重点实验室, 北京 102205; State Key Laboratory of NBC Protection for Civilian, Beijing 102205, China

**Keywords:** 高效液相色谱-串联质谱, 季铵盐, 维库溴铵, 罗库溴铵, 泮库溴铵, 肌松剂, high performance liquid chromatography-tandem mass spectrometry (HPLC-MS/MS), quaternary ammonium, vecuronium, rocuronium, pancuronium, muscle relaxants

## Abstract

维库溴铵、罗库溴铵和泮库溴铵是一类广泛使用的非去极化肌松剂,使用过程中引起过敏反应甚至死亡的情况时有发生,快速检测血液中该类肌松剂的浓度,可为临床早期诊断提供有价值的信息。该类肌松剂为强极性的季铵盐类化合物,在反相色谱柱上难以保留,主要采用离子对色谱法进行分离。采用离子对色谱法时,加入的离子对试剂有离子抑制作用,可降低质谱灵敏度,还会污染质谱系统。该文建立了高效液相色谱-串联质谱(HPLC-MS/MS)同时检测血液中3种季铵盐类肌松剂的检测方法。血液样品经稀释、高速离心后,上清液过Bond Elut AL-N固相萃取柱净化,用0.45 μm的微孔滤膜过滤后检测。采用ZIC-cHILIC色谱柱(50 mm×2.1 mm, 3.0 μm)分离,以乙腈和0.1%甲酸水溶液为流动相,梯度洗脱,在ESI^+^、多反应检测(MRM)模式下检测。3种季铵盐类肌松剂在血液中的基质效应为88.1%~95.4%,在各自范围内线性关系良好,相关系数(*R*^2^)均大于0.996,检出限为0.2~0.8 ng/mL,定量限为0.5~2.0 ng/mL,加标回收率为92.8%~110.6%,相对标准偏差(RSD)为3.2%~9.4%。该方法灵敏度高,准确性好,操作简便,可用于血液样品中维库溴铵、罗库溴铵和泮库溴铵的快速检测。

## Abstract (en)

Vecuronium, rocuronium, and pancuronium are widely used as non-depolarizing muscle relaxants. There have been occasional cases of allergic reactions and even death when using such muscle relaxants. Rapid determination of the concentration of these muscle relaxants in blood can provide valuable information for early clinical diagnosis. As quaternary ammonium compounds, these muscle relaxants are highly polar. Hence, they cannot be retained effectively on reversed-phase chromatographic columns with conventional mobile phases. These quaternary ammonium muscle relaxants are mainly separated by ion-pair chromatography. Using an ion-pairing reagent can help improve the retention capabilities of quaternary ammonium muscle relaxants. Nevertheless, the sensitivity of MS detection is significantly decreased because of ionic inhibition caused by the ion-pairing reagent in the mobile phase. Furthermore, ion-pairing reagents can pollute the MS system. A method based on high performance liquid chromatography-tandem mass spectrometry (HPLC-MS/MS) was established for the simultaneous determination of the three quaternary ammonium muscle relaxants in blood. The blood samples were diluted and subjected to high-speed centrifugation. The supernatant was purified on a Bond Elut AL-N solid phase extraction column and then filtered through a 0.45 μm microporous membrane. The quaternary ammonium muscle relaxants were separated on a ZIC-cHILIC analytical column (50 mm×2.1 mm, 3.0 μm) with gradient elution. Acetonitrile and 0.1% formic acid aqueous solution were used as mobile phases. The separated compounds were analyzed by tandem MS with an electrospray ionization (ESI) source in positive and multiple reaction monitoring (MRM) modes. The matrix effects of vecuronium, rocuronium, and pancuronium in blood were 88.1% to 95.4%. The calibration curves for vecuronium, rocuronium, and pancuronium showed good linear relationships in each range, and all correlation coefficients (*R*^2^) were > 0.996. The limits of detection of vecuronium, rocuronium, and pancuronium were 0.2-0.8 ng/mL, with the corresponding limits of quantification being 0.5-2.0 ng/mL. The recoveries of vecuronium, rocuronium, and pancuronium were 92.8% to 110.6%, with relative standard deviations (RSDs) of 3.2%-9.4%. This method is sensitive, accurate, and easy to operate, and it can be used to rapidly determine vecuronium, rocuronium, and pancuronium in blood.

维库溴铵(vecuronium bromide)、罗库溴铵(rocuronium bromide)和泮库溴铵(pancuronium bromide)是甾类非去极化肌松剂,与其他非去极化肌松剂相比,具有起效作用快、作用时间长、体内无积聚、安全性高等优点,被广泛应用于气管插管和外科手术中^[1,2,3,4]^。但在使用过程中,该类肌肉松弛剂可引起红斑疹、恶心、呕吐、头晕等过敏反应,甚至死亡^[5,6,7,8]^,建立快速定量分析该类肌松剂的方法,对临床诊断、产品质量控制和安全用药等均有重要应用价值。

维库溴铵、罗库溴铵和泮库溴铵均是季铵盐结构,为强极性水溶性化合物,在反相色谱柱上难以保留,多采用离子对色谱法进行分离。目前,检测季铵盐类肌松剂的方法主要有液相色谱法(HPLC)^[9,10,11,12,13,14,15]^、液相色谱-串联质谱法(LC-MS/MS)^[16,17,18,19,20,21]^和毛细管电泳法(CZE)^[20,21]^等。其中,HPLC不适于痕量检测,其流动相主要为离子对试剂,流动相组成相对复杂,不同文献报道的流动相组成也有所不同。离子对试剂有离子抑制作用,会降低质谱检测的灵敏度,无法将应用于HPLC的流动相直接应用于LC-MS/MS。已报道的检测肌松剂的LC-MS/MS也存在一些不足,如加标回收率范围较宽^[22]^、检出限较高^[23]^、测定时间较长^[24]^等。

本文采用HPLC-MS/MS对血液中3种季铵盐类肌松剂进行了定量分析。血液样品经稀释、离心和固相萃取柱净化,经色谱柱分离后进入质谱检测。该方法前处理简便,测定时间短,检出限低,回收率较好,能够满足血液样品中3种季铵盐类肌松剂同时检测的要求。

## 1 实验部分

### 1.1 仪器、试剂与材料

Agilent 1200-6410 Triple Quad高效液相色谱-质谱联用仪、0.45 μm微孔滤膜(美国Agilent公司); Bond Elut AL-N固相萃取柱(60 mg,美国Varian公司); XP105天平(上海Mettler-Toledo公司)。

乙腈(HPLC级,德国Merk KGaA公司);甲酸、三氟乙酸和乙酸铵(分析纯,上海Macklin Biochemical公司);维库溴铵、罗库溴铵和泮库溴铵(纯度均>95%,北京百灵威科技有限公司);实验用水为超纯水。

### 1.2 标准溶液的配制

标准储备溶液:分别准确称取适量的维库溴铵、罗库溴铵和泮库溴铵样品,用乙腈溶解并定容,配制成1 mg/mL的标准储备液,室温避光保存。

标准工作溶液:分别取上述标准储备溶液适量,用乙腈定容至10 mL,配制成已知浓度的混合标准工作溶液,室温避光保存。

### 1.3 样品前处理

取血液样品0.5 mL,加入甲酸-水-乙腈(2:48:50, v/v/v)溶液4 mL,振荡均匀,以5000 r/min离心30 min,取上清液过Bond Elut AL-N固相萃取柱(先用3 mL甲酸-水-乙腈(2:48:50, v/v/v)溶液活化),精密吸取3 mL甲酸-水-乙腈(2:48:50, v/v/v)洗脱目标物,并用0.45 μm的微孔滤膜过滤,待用。

### 1.4 仪器条件

1.4.1 色谱条件

色谱柱:ZIC-cHILIC柱(50 mm×2.1 mm, 3.0 μm,德国Merck KGaA公司);柱温:30 ℃;流动相A:乙腈;流动相B: 0.1%甲酸水溶液。梯度洗脱程序:0~0.8 min, 70%A; 0.8~0.9 min, 70%A~50%A; 0.9~1.3 min, 50%A~40%A; 1.3~1.4 min, 40%A; 1.4~1.8 min, 40%A~10%A; 1.8~1.9 min, 10%A~70%A; 1.9~3.0 min, 70%A。进样量5 μL。

1.4.2 质谱条件

电喷雾电离(ESI)源,正离子扫描模式;毛细管电压4000 V;脱溶剂气温度300 ℃;脱溶剂气流量8 L/min;多反应监测(MRM)模式。各待测物的定性及定量离子对、锥孔电压和碰撞能量等参数见表1。

**表 1:** 3种肌松剂的质谱参数

Compound	Precursor ion	Mass transition (m/z)	Fragmentor/ V	Collision energy/ eV
Vecuronium	[M-Br^-^+H]^2+^	279.2/100.1	131	24
bromide		279.2/249.2^*^		8
Rocuronium	[M-Br^-^+H]^2+^	265.2/235.1	122	12
bromide		265.2/244.2^*^		9
Pancuronium	[M-2Br^-^]^2+^	286.6/100.1^*^	123	21
bromide		286.6/256.2		10

* Quantification ion pair.

## 2 结果与讨论

### 2.1 质谱条件优化

取1.0 μg/mL 3种目标物的标准溶液,分别以自动进样的方式在ESI^+^模式下进行质谱条件优化,3种目标物全扫描质谱图见图1。结果表明,单电荷离子结构的维库溴铵和罗库溴铵均会产生[M-Br^-^]^+^和[M-Br^-^+H]^2+^,其*m/z*分别为557.4、279.2和529.4、265.2,双电荷离子结构的泮库溴铵则产生*m/z*为286.6的[M-2Br^-^]^2+^。最优锥孔电压下,维库溴铵和罗库溴铵[M-Br^-^+H]^2+^的丰度均高于[M-Br^-^]^+^的丰度,故选用维库溴铵和罗库溴铵[M-Br^-^+H]^2+^、泮库溴铵[M-2Br^-^]^2+^为母离子进行二级质谱条件优化。通过子离子扫描得到目标物碎片离子信息,并对碰撞能量进行优化,每个目标物以响应最高的分子离子对作为定量离子对,响应次高的分子离子对作为定性离子对(见表1)。

**图 1:**
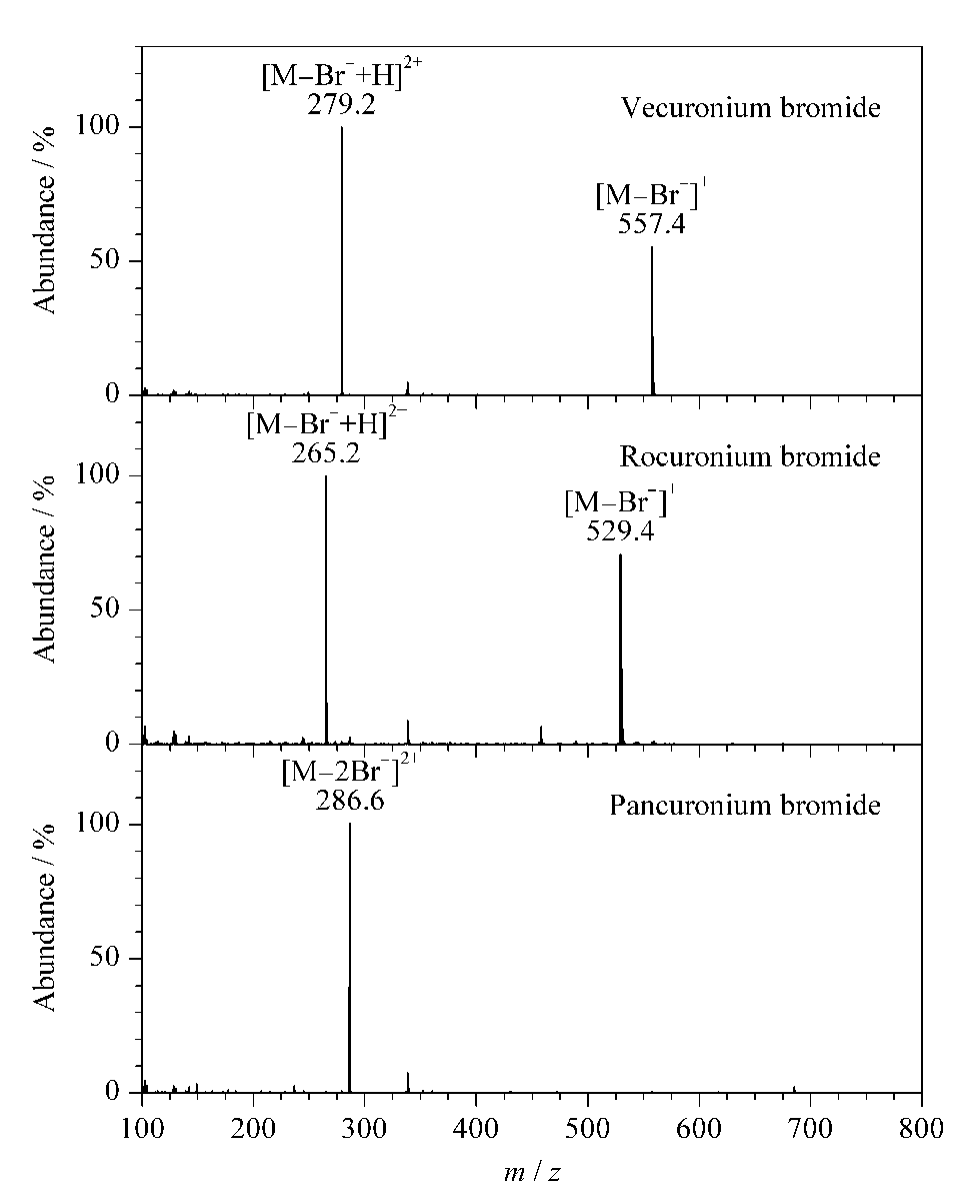
3种肌松剂的全扫描质谱图

### 2.2 色谱条件优化

由于维库溴铵、罗库溴铵和泮库溴铵均为季铵盐类化合物,色谱柱类型和流动相组成均会对其保留时间、色谱峰形以及离子化效率产生影响,并最终影响目标物的检测灵敏度。

实验在保持3个目标物浓度、流速、进样量等参数一致,且流动相为乙腈-0.1%甲酸水溶液(80:20, v/v)的条件下,分别考察了美国Agilent公司的SB-C8(50 mm×4.6 mm, 1.8 μm)、SB-C18(50 mm×4.6 mm, 1.8 μm)、ZORBAX HILIC Plus(50 mm×2.1 mm, 3.5 μm)和德国Merck KGaA公司的ZIC-HILIC(50 mm×2.1 mm, 3.0 μm,)、ZIC-cHILIC(50 mm×2.1 mm, 3.0 μm)等5种色谱柱对目标物色谱峰形、离子化效率和保留时间的影响(见图2)。结果表明,采用SB-C8和SB-C18色谱柱时,20 min内没有出现目标物色谱峰,这是由于3种带正电荷的肌松剂极易被硅胶基色谱柱上残留的硅羟基吸附,需在流动相中加入较高浓度的缓冲盐或离子对试剂才能减少吸附作用,而高浓度的缓冲盐和离子对试剂对质谱信号有抑制作用,且会对质谱系统产生不利影响^[25]^;采用ZORBAX HILIC Plus色谱柱时,虽然色谱峰对称性较好,但目标物的色谱峰丰度均较低,不利于提高检测灵敏度;采用ZIC-HILIC和ZIC-cHILIC色谱柱时,色谱峰均有较好的对称性,但在ZIC-cHILIC柱上的色谱峰丰度更高,保留效果也更好。因此选用ZIC-cHILIC为实验色谱柱。

**图 2:**
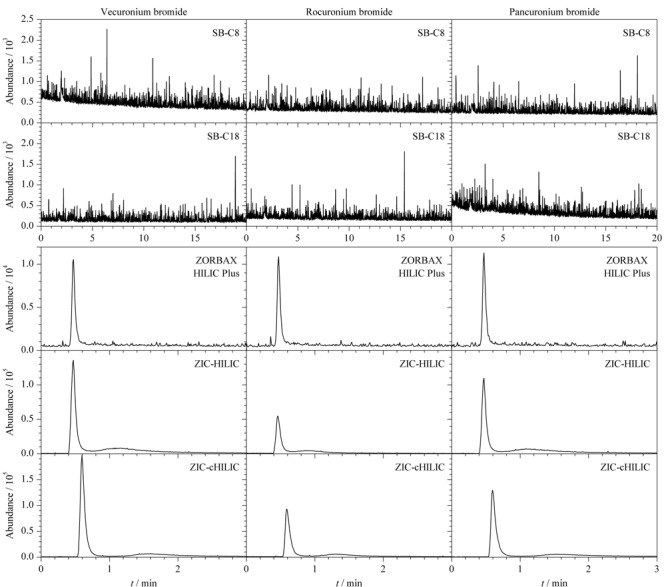
不同色谱柱上3种肌松剂的提取离子流色谱图

实验同时考察了5 mmol/L乙酸铵水溶液,0.1%三氟乙酸水溶液和0.1%甲酸水溶液等常用水相对目标物的离子化效率的影响(见图3)。实验中保持3个目标物浓度、流速、进样量等参数一致,流动相中的有机相乙腈的体积分数为80%,色谱柱为ZIC-cHILIC柱。结果表明,以0.1%甲酸水溶液为水相时,3个目标物的离子化效率和保留效果均优于其他2种溶液,故选择0.1%甲酸水溶液为流动相中的水相。

**图 3:**
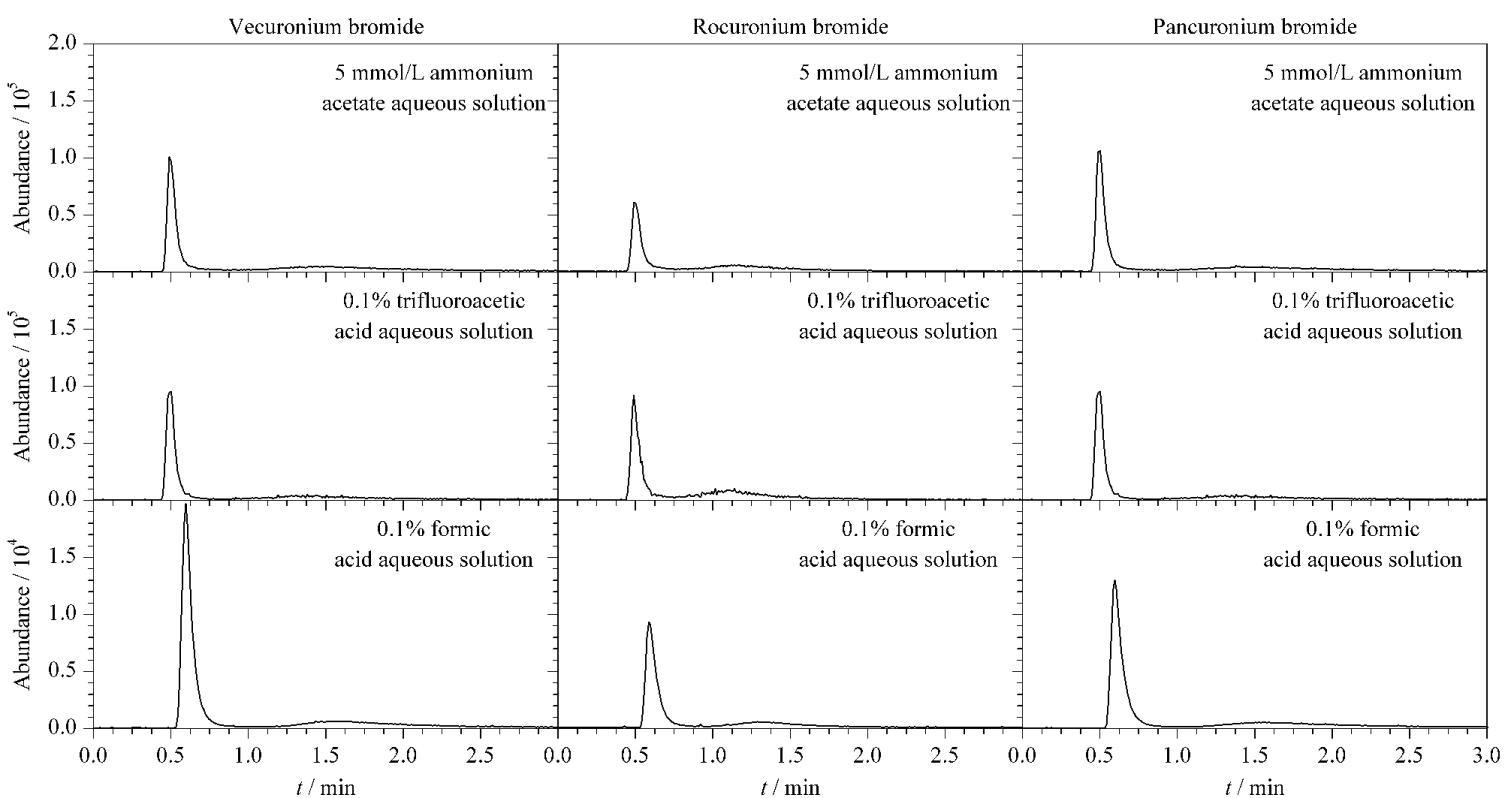
采用不同流动相时3种肌松剂的提取离子流色谱图

实验以乙腈和0.1%甲酸水溶液为流动相,采用优化后的梯度洗脱程序,对3种季铵盐类肌松剂混合标准溶液进行分析,由其总离子流色谱图(见图4a)可以看出,3种季铵盐类肌松剂在3 min内可较好的分离。

**图 4:**
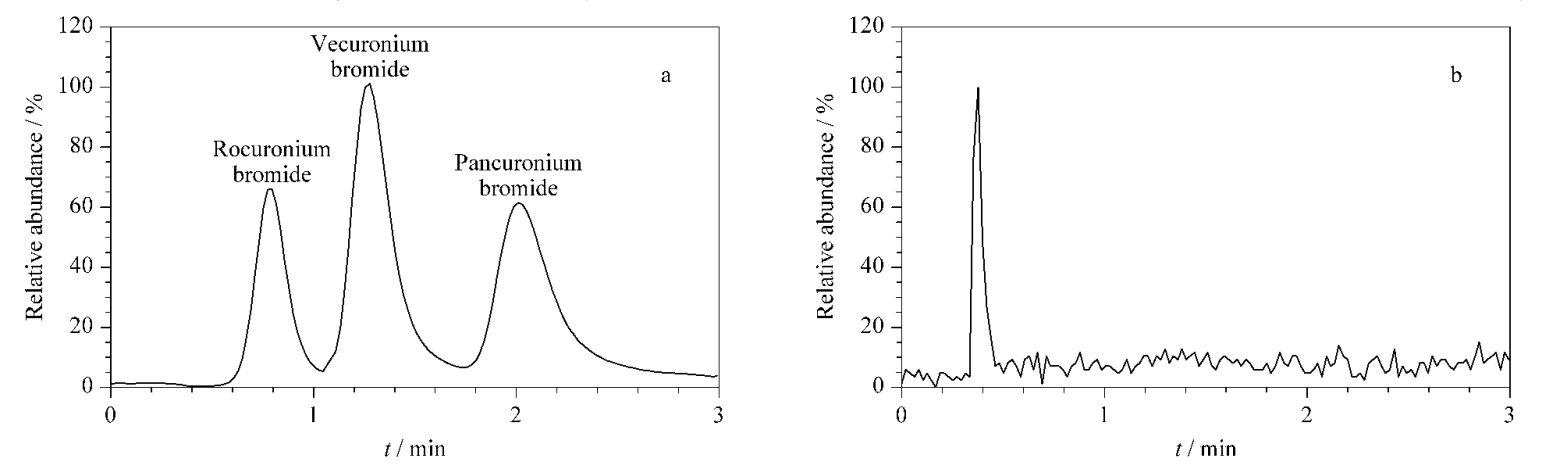
(a)3种肌松剂混合标准溶液和(b)空白血液样品的总离子流色谱图

### 2.3 样品处理条件的选择

考察了不同类型的固相萃取柱对目标物的净化效果,包括OASIS MCX 3cc离子交换固相萃取柱(60 mg,美国Waters公司)、Carboxylic Acid固相萃取柱(50 mg,美国J. T. Baker公司)和Bond Elut AL-N固相萃取柱(60 mg,美国Varian公司)。

参照文献^[22]^方法,血液样品先经稀释、离心,然后取上清液过OASIS MCX 3cc离子交换固相萃取柱,以甲酸-乙腈-水(2:48:50, v/v/v)为洗脱剂对血液样品进行净化,每次用1 mL洗脱剂进行洗脱并进行检测,结果显示,5 mL内很难将目标物完全洗脱,其回收率低于60%;参照上述方法,采用同样的洗脱剂,使用Carboxylic Acid固相萃取柱对血液样品进行净化,结果显示,目标物与填料结合作用较强,5 mL内几乎没有目标物被洗脱;采用同样洗脱剂,使用Bond Elut AL-N固相萃取柱净化时,前2 mL洗脱液中3种目标物含量均大于97%,第4 mL洗脱液中无法检测到目标物。因此,实验采用Bond Elut AL-N固相萃取柱,以3 mL的甲酸-乙腈-水(2:48:50, v/v/v)为洗脱剂,对血液样品进行净化处理。

### 2.4 方法学考察

2.4.1 专属性和基质效应

将0.5 mL空白血液样品采用1.3节方法进行前处理,然后进样测定,其总离子流色谱图见图4b。结果表明,空白血液样品中内源性物质对3种目标物的测定不产生干扰,方法专属性良好。

在空白血液的前处理液中加入3种目标物,分别配制50 μg/L和100 μg/L混合基质标准溶液,进样分析,得到目标物的峰面积(*A*),同时测定相同水平的混合标准溶液,得到目标物的峰面积(*B*)。根据公式ME=*A/B*×100%计算基质效应。结果表明,空白血液中维库溴铵、罗库溴铵和泮库溴铵的ME值分别为89.5%~94.2%、88.1%~92.8%和90.6%~95.4%,基质效应较弱,可以使用溶剂标准曲线进行定量。

2.4.2 标准曲线、检出限和定量限

采用1.4节的仪器条件对1.2节配制的标准工作溶液进行测定。以各分析物的峰面积(*Y*)和对应的质量浓度(*X*, ng/mL)进行线性回归,得到3种目标物的标准曲线和相关系数(*R*^2^),以特征离子色谱峰*S/N* ≥ 3和10时目标物的含量为方法的检出限和定量限(见表2)。结果表明,目标物在对应的浓度范围内线性关系良好,相关系数均大于0.996,检出限为0.2~0.8 ng/mL,定量限为0.5~1.7 ng/mL。

**表 2:** 3种肌松剂的线性范围、线性方程、相关系数、检出限、定量限、回收率及相对标准偏差

Compound	Linear range/ (ng/mL)	Linear equation	R^2^	LOD/ (ng/mL)	LOQ/ (ng/mL)	Recovery/%	RSD (n=6)/%
Vecuronium bromide	0.5-1000	Y=79.68X+945.47	0.9967	0.2	0.5	92.8-108.3	3.2-5.6
Rocuronium bromide	2.0-1000	Y=107.36X-3040.50	0.9972	0.8	2.0	94.3-106.2	4.2-5.4
Pancuronium bromide	1.7-1000	Y=88.68X-1881.70	0.9963	0.7	1.7	97.9-110.6	5.5-9.4

*Y*: peak area; *X*: mass concentration, ng/mL.

2.4.3 加标回收率和精密度

在空白血液中分别添加4个水平(定量限、3倍定量限、200 ng/mL和500 ng/mL)的目标物,加标样品按照1.3节方法处理后进行测定,得到3种目标物的平均回收率为92.8%~110.6%,相对标准偏差为3.2%~9.4%(*n*=6)(见表2)。该方法能够较好地满足血液样品中3种肌松剂含量的测定要求。

### 2.5 实际样品的测定

采用本方法对4个实际兔子血液样品进行测试,每个样品均采用1.3节的方法进行前处理,结果表明,样品中维库溴铵、罗库溴铵和泮库溴铵的含量分别为231~364、173~450和287~428 ng/mL(见表3),均在本方法的线性范围内。

**表 3:** 4个兔子血液样品的测定结果(*n*=3)

No.	Vecuronium bromide/(ng/mL)	Rocuronium bromide/(ng/mL)	Pancuronium bromide/(ng/mL)
1	ND	ND	428
2	364	ND	ND
3	ND	450	ND
4	231	173	287

ND: not detected.

## 3 结论

本文通过对色谱柱、流动相、质谱测定条件以及血液样品前处理条件的优化,建立了HPLC-MS/MS同时测定血液中维库溴铵、罗库溴铵和泮库溴铵的分析方法,并应用于实际样品的检测。该方法前处理简便,测定时间短,线性范围宽,检出限低,灵敏度高,为血液样品中3种季铵盐类肌松剂的同时测定提供了简单、实用的方法。
